# Antimicrobial therapies for prevention of recurrent acute exacerbations of COPD (AECOPD): beyond the guidelines

**DOI:** 10.1186/s12931-022-01947-5

**Published:** 2022-03-14

**Authors:** Michelle Brennan, M. J. McDonnell, M. J. Harrison, N. Duignan, A. O’Regan, D. M. Murphy, C. Ward, R. M. Rutherford

**Affiliations:** 1 Department of Respiratory Medicine, Galway University Hospital, Newcastle Road, Galway, Ireland; 2 Department of Respiratory Medicine, Cork University Hospital, Galway, Ireland; 3 Department of Translational and Clinical Research Institute, Faculty of Clinical Sciences, University of Newcastle Upon Tyne, Newcastle Upon Tyne, UK

## Abstract

**Background:**

Unfortunately, many COPD patients continue to exacerbate despite good adherence to GOLD Class D recommended therapy. Acute exacerbations lead to an increase in symptoms, decline in lung function and increased mortality rate. The purpose of this review is to do a literature search for any prophylactic anti-microbial treatment trials in GOLD class D patients who ‘failed’ recommended therapy and discuss the role of COPD phenotypes, lung and gut microbiota and co-morbidities in developing a tailored approach to anti-microbial therapies for high frequency exacerbators.

**Main text:**

There is a paucity of large, well-conducted studies in the published literature to date. Factors such as single-centre, study design, lack of well-defined controls, insufficient patient numbers enrolled and short follow-up periods were significant limiting factors in numerous studies. One placebo-controlled study involving more than 1000 patients, who had 2 or more moderate exacerbations in the previous year, demonstrated a non-significant reduction in exacerbations of 19% with 5 day course of moxifloxacillin repeated at 8 week intervals. In *Pseudomonas aeruginosa* (*Pa*) colonised COPD patients, inhaled antimicrobial therapy using tobramycin, colistin and gentamicin resulted in significant reductions in exacerbation frequency. Viruses were found to frequently cause acute exacerbations in COPD (AECOPD), either as the primary infecting agent or as a co-factor. However, other, than the influenza vaccination, there were no trials of anti-viral therapies that resulted in a positive effect on reducing AECOPD. Identifying clinical phenotypes and co-existing conditions that impact on exacerbation frequency and severity is essential to provide individualised treatment with targeted therapies. The role of the lung and gut microbiome is increasingly recognised and identification of pathogenic bacteria will likely play an important role in personalised antimicrobial therapies.

**Conclusion:**

Antimicrobial therapeutic options in patients who continue to exacerbate despite adherence to guidelines-directed therapy are limited. Phenotyping patients, identification of co-existing conditions and assessment of the microbiome is key to individualising antimicrobial therapy. Given the impact of viruses on AECOPD, anti-viral therapeutic agents and targeted anti-viral vaccinations should be the focus of future research studies.

## Introduction

The Global Initiative for Chronic Lung Disease (GOLD) annual report is the most commonly used COPD guideline in clinical practice and is based on the appraisal of the best current scientific evidence [1]. Whilst evidence-based guidelines can standardise and improve care for a number of patients, the margins of benefit, even in responders, may not be that substantial. COPD is a complex and heterogeneous conditions, a generalised approach to treatment options will result in a medley of outcomes for patients; some may find treatment side-effects intolerable, some may not respond; and, unfortunately, some patients may experience inequitable access to certain treatments recommended.

COPD accounts for a huge burden in terms of health care costs and early mortality. Patients who exacerbate more frequently have a lower quality of life, a more rapid decline in their FEV1 and have a higher mortality [2]. A history of previous exacerbation events is the single largest risk factor for predicting future exacerbations [3]. Multiple risk factors for exacerbations have been shown and many patients will have several predisposing conditions or environmental factors, see Table 1 [4–12].

**Table 1:** Co-factors in acute exacerbations of COPD

Reduced mucociliary clearance	GORD	Airway colonization	Immune deficiency	Aspiration	GOLD D treatment
Direct injury by tobacco smoke	Increased frequency of Hiatus Hernia [4] and GORD [6] leading to chemical, food and microbial aspiration	*H Influenzae* *S pneumonia* *M catarrhalis* *P aeruginosa*	Innate immunity impaired- shortening of cilia, squamous cell metaplasia, goblet-cell hyperplasia, loss of tight junction from toxic effects of smoking	Swallowing normally performed in exhalation. In COPD pts swallowing can be immediately before or after inspiration heightening aspiration -risk considerably [12]	•LAMA/LABA •ICS •Azithromycin •Roflumilast •Influenza Vacc •Pneumococcal Vacc •Pulmonary rehabilitation within 6 weeks of hospital discharge for AECOPD
Chronic airway inflammation ± bronchiectasis		*Adenovirus* *Influenza B* *Coronovirus* *Rhinovirus* *Influenza A* [6, 7]	Adaptive immunity- fewer CD4 + T central memory cells and CD8 + T effector memory cells [8]		
Recent exacerbation			Primary Immune deficiency disease-hypogammaglobulinaemia, specific antibody deficiency, selective IgA deficiency [9]		
Airways obstruction			Immunosenescence- cellular senescence, stem cell exhaustion, increased oxidative stress, alteration in extracellular matrix, reduction in endogenous anti-ageing molecules [10]		
Dynamic expiratory collapse			Supressed antiviral immune response [11]		
↑Mucus tenacity					
Expiratory muscle weakness—sarcopenia, altered pulmonary dynamics					

Specific preventative anti-microbial therapy in the GOLD D guidelines only consists of one antibiotic, azithromycin, and two vaccinations [1]. When compared with the complex milieu of potential insults triggering exacerbations, the GOLD D treatment certainly appears suboptimal given the absence of proven prophylactic anti-exacerbation therapies. Azithromycin reduces exacerbations by about 30% in patients exacerbating in the previous year or in those on long-term oxygen therapy [13].

Given that many GOLD Class D patients fail to respond to current guideline-directed GOLD recommended care, it is inevitable that clinicians will try other strategies in an attempt to prevent exacerbations. Significant benefits of therapeutic interventions are difficult and challenging to show in even well-conducted RCTs of COPD populations. Given the complexity encountered and inter-patient differences within COPD populations, a negative study does not necessarily preclude a small number of patients with specific characteristics having a significant benefit. For the purposes of this article, we have chosen to concentrate on antimicrobial strategies in GOLD D patients with refractory exacerbations. A search strategy was performed on PUBMED using the terms ‘Recurrent Exacerbations’, ‘COPD’, ‘Antimicrobial therapies’ and ‘Microbiome’ was performed. Relevant studies that described pathophysiology, microbiome studies and treatment regimes were chosen for discussion.

### Phenotypes in COPD

COPD is a complex and heterogenous condition that involves numerous pathological processes resulting in a number of subgroups that have their own characteristics and natural history of disease [14, 15]. In lieu of a one-size-fits all approach to therapy, antimicrobial agents must be tailored to individual patient or subgroup characteristics and prophylactic or treatment medicines considered for those who are most likely to benefit and least likely to be harmed by this approach.

Burgel et al., recently reported that the GOLD guidelines do not take into account the impact of factors such as age, co-morbidities and BMI when assigning severity classes to patients [16]. Their study employing cluster analysis, in a derivation cohort, described five phenotypes in 2409 patients with COPD. They include the factors listed above in addition to functional assessments and FEV1 to classify patients into five subgroups ranging from ‘mild respiratory with a 3 year mortality rate of only 2.5% to ‘the severe comorbid’ with frequent exacerbations and a 3-year mortality of over 50%. An approach to antimicrobial therapies in the most severe classes should take into account concurrent diseases, a higher likelihood of resistant organisms and potential colonisation, rather than strict adherence to standard community LRTI guidelines in the event of AECOPD.

Additionally, Spanish guidelines for COPD management proposed four different clinical COPD phenotypes—(1) a ‘non-exacerbation type’ (2) ‘asthma/COPD overlap type’ (3) ‘frequent exacerbator with emphysema type’ and (4) ‘exacerbator with chronic bronchitis type’ [17].

Physicians should consider which subgroup their patient belongs to and consider evidence-based management for exacerbation prevention and antimicrobial therapies.

Management of ‘non-exacerbator’ phenotype focuses on dual bronchodilator therapy and antimicrobial prophylaxis is unlikely to be necessary. In those with asthma/COPD overlap syndrome should be prescribed inhaled corticosteroids as part of their regime. Similar to patients with severe refractory asthma the ‘asthma/COPD overlap type’ with eosinophilia have been shown to benefit from monoclonal antibody targeted against Interleukin-5 [18]. Mepolizumab has been trialled and was found to be associated with lower rates of moderate or severe exacerbations compared with placebo [19]. It is pertinent to identify those patients with bronchial chronic obstruction who can benefit from treatment with ICSs, and, in a hypothetical near future, from additional biological drugs.

In patients with ‘frequent exacerbation with emphysema’ and ‘exacerbator with chronic bronchitis’ a thorough evaluation for co-existing bronchiectasis, GORD and sinus disease should be performed.

The prevalence of bronchiectasis ranges from 4 to 72% in patients with severe COPD depending on the study methodology [20]. A high-resolution CT (HRCT) with expiratory images, is critical to further management of this group. This is to confirm the presence of bronchiectasis, evaluate the extent and location and identify mucus plugging, Patients with confirmed bronchiectasis should be referred for PEEP-based physiotherapy and airway clearance education. Sputum cultures are vital for direct antibiotic choice and patients will require extended courses of antibiotics for 10 to 14 days in the event of an exacerbation. Long-term macrolide treatment significantly reduces the frequency of exacerbations in patients with bronchiectasis and should also be considered for patients with recurrent exacerbations in COPD-Bronchiectasis overlap [21].

Microbes play a significant role in the pathogenesis and progression of COPD and bronchiectasis, particularly *Haemophilus* and *Pa*. Microbial dysbiosis associates with more severe disease and frequent sputum surveillance is required to determine if they been colonised with resistant organisms such as *Pa* or *Stenotrophomonas maltophilia (Sm)* [22].

Patients with symptoms suggestive of GORD or aspiration should undergo an evaluation by speech and language therapy and regular dental review to assess for evidence of periodontitis as a provoking factor for exacerbations [12, 23].

Co-existing obstructive sleep apnoea (OSA) is also associated with increased exacerbation frequency and more severe exacerbations [24]. Treatment with continuous positive airway pressure (CPAP) confers a significant benefit in OSA-COPD overlap patients with a ?observational cohort study by Marin et al. demonstrating a relative risk of hospitalisation with AECOPD of 1.7 times compared with untreated patients [25]. A further matched cohort study of OSA-COPD patients with AECOPD compared to those without an exacerbations history found that worse airflow limitation and lower CPAP usage were strong predictors of COPD exacerbations and reduced survival independently of coexisting cardiovascular and metabolic disorders [26]. The disease mechanism is unclear but forced inspirations against a closed upper airway may siphon gastric contents up to the larynx and then into the trachea. Patients with OSA are likely to exhibit an impaired swallowing reflex, probably due to the perturbed neural and muscular function of the upper airways. This suggests patients with OSA may perturb the inspiratory-expiratory transition during deglutition in patients, resulting in increased vulnerability to aspiration [27].

### Evidence for infectious aetiology in AECOPD

Respiratory tract infections are well recognized as triggers of acute exacerbations of COPD leading to a vicious cycle of infection and inflammation and progressive airway damage [28]. A 2020 systemic review showed the prevalence of bacterial infections is about 50%, although 20% may be a co-infection of simultaneous bacterial and viral infection [29]. The role of bacterial pathogens including *Haemophilus influenzae*, *Streptococcus pneumoniae*, and *Moraxella catarrhalis*, is well established and their prevalence justifies the rationale for empirical antibiotic therapy in patients with evidence of an infective exacerbation. The role of viruses in AECOPD is increasingly recognised and is the focus of recent studies.

An observational cohort study by George et al., assessed the association between sputum PCR and patient reported exacerbations. HRV prevalence and viral load at the time of exacerbations was higher than during stable states (p < 0.001) and patients who had HRV detected at exacerbations had higher median exacerbation frequency than those without HRV (p = 0.038) [30]. Other viruses detected at a lower frequency in observational cohort studies include parainfluenza virus 3 (0.6–7%), coronaviruses (1–9%), adenovirus (0.6–7%) and human metapneumovirus (1–8%) [6]. A systematic review of worldwide prevalence of viral infection in AECOPD reported a pooled prevalence of 37% with HRV being the most common and echovirus the least common, fluctuations in prevalence was notable between years [31].

Co-infections with two or more viruses or viruses and bacteria have been reported in up to 25% of all AECOPD in observational studies [32–34]. It remains unclear what impact dual infection has on the severity of exacerbations as conflicting evidence on outcomes has been reported thus far.

The main strategies that are recognised to combat viral infections to date are a prophylactic approach, by vaccination and immunoprophylaxis. The heterogeneity of viruses, for example HRV, makes targeted immunoprophylaxis difficult to achieve in clinical practice [35, 36]. Another approach is employing the use of small molecule inhibitors [37]. There are currently no studies evaluating the role of anti-viral therapies in COPD populations, this is an important consideration given the prevalence of viral infections as the primary or concurrent aetiology of AECOPD and future research is urgently needed to address this.

The role of antiviral agents has been evaluated in non-COPD populations. Passive immunoprophylaxis against RSV using palivizumab has been trialled in high risk infants [38]. In 2009, a nine-day treatment regime of vapendavir was found to reduce viral load in healthy volunteers infected with HRV [39]. In 2017, phase 2b clinical trial (SPIRITUS) reported vapendavir was associated with a statistically significant antiviral effect if administered to asthmatic patients within 24 h of symptom onset [40].

As upper respiratory tract symptoms may precede AECOPD in up to 4% of cases, thus reducing rhinovirus infections may offset an acute exacerbation if treatment is initiated promptly [41]. The use of highly potent antivirals, may offer a more realistic strategy to reduce the burden of rhinovirus infections in COPD patients than immunoprophylaxis. Antiviral drugs would ideally possess broad spectrum activity against the heterogeneity encountered within HRV groups in order to be maximally effective [42].

A recent observational cohort study in Hong Kong reported a reduction of 44% in number of patients hospitalised with acute exacerbations of COPD during the first three months of 2020 compared to the previous 5 years [43]. The authors postulated that universal masking and social distancing could have prevented respiratory tract infections and hence resulted in decrease in AECOPD. This is further supported by a study performed in Singapore which reported significant reduction in AECOPD admissions and statistically significant reduction in PCR-positive respiratory viral infections from 48% pre-pandemic to 10.8% during pandemic [44]. In addition to pharmaceutical therapies, an emphasis on conservative anti-infection strategies as adopted for the COVID-19 pandemic should be encouraged, particularly during seasonal outbreaks.

## Antimicrobial therapies

### Pulsed fluroquinolones

The double blind, placebo-controlled RCT ‘ PULSE study’ included over 1000 patients with COPD and a history of ≥ 2 moderate exacerbations in the previous year without colonisation with pathogens resistant to moxifloxacin. Participants were randomised to either moxifloxacin 400 mg given once daily for 5 days and repeated every 8 weeks for a total of six courses, or placebo [45]. Patients were followed up for 6 months post end of treatment. In the intention-to-treat analysis, pulsed therapy with moxifloxacin reduced the odds of an exacerbation by 19% in patients with moderate to severe COPD, which was not statistically significant (p = 0.059). However, a post-hoc subgroup of analysis of those with mucopurulent/purulent sputum there was 15.2% absolute risk reduction in exacerbations and an NNT of 7 (p = 0.004). The safety outcomes reported no increase in rates of bacterial resistances to antibiotics, and no serious adverse events due to the treatment particularly, cardiac or *Clostridium difficile* infection.

### Doxycycline

Anecdotally, doxycycline is a common second line choice for antimicrobial prophylaxis in patients who have contra-indications to macrolide antibiotics or continue to exacerbate while taking azithromycin. Despite widespread use in clinical practice, there is a paucity of studies to support its merits in prophylaxis. A single-blind, randomised placebo-controlled trial by Brill et al., in 2015 evaluated three therapeutic interventions; pulsed moxifloxacin 500 mg once daily for 5 days every month, azithromycin 250 mg three times weekly and doxycycline 100 mg daily compared to placebo in 99 patients with moderate to severe COPD [46].Total airway bacterial load did not decrease significantly, measured using 16S PCR and airways inflammation, in any of the intervention groups at 3 month follow up. Moreover, increases in antibiotic resistance were seen in all treatment groups.

Shafuddin et al., performed a double-blind, placebo-controlled RCT which recruited 292 patients with high frequency exacerbator phenotype of COPD [47]. Patients were randomised to either roxithromycin 300 mg and doxycycline 100 mg daily, roxithromycin only or placebo for a period of 12 weeks. At follow up there no difference in exacerbations rates with either of the treatment arms in comparison to placebo. In addition, there was no difference in spirometry or quality of life scores at follow up. A recent RCT randomised patients with at least moderate COPD and at least 1 exacerbation in the previous year to either doxycycline 100 mg daily or placebo for 12 months. In total, 222 patients were included, and one- third were current smokers. Overall, doxycycline was not found to reduce the exacerbation rate based on patient reported symptoms or additional medication usage. The response varied according to baseline eosinophil account with lower serum eosinophil counts associated with a clinical benefit [48].

## Inhaled antibiotics (Table 2)

**Table 2:** Summary of inhaled antibiotic regimes in COPD

Antibiotic	Author and year	Dosing and duration	Study design and population	Study results
Tobramycin	Del Negro et al. 2008 [53]	300 mg BD via nebuliser for 14 days Follow up 6 months	Prospective Auto-control cohort study N = 13 Severe COPD and colonized with multi drug resistant *P aeruginosa*	Reduction in interleukin-1B, IL-8 and eosinophils *P aeruginosa* density lower 42% reduction in exacerbations
Colistin	Bruguera-Avila et al 2017 [54]	1 ml BD via nebuliser for at least 3 months Follow up 12 months	Prospective auto-control cohort study N = 36 with COPD and *Pa* colonisation	Reduction in admissions Reduced Length of stay for hospitalisations
Colistin and continuous cyclic azithromycin	Montoin et al 2019 [55]	Colistin either 1–2 million IU BD or 0.5–1 million IU BD depending on drug and nebuliser device Minimum 3 months Azithromycin 500 mg three times weekly PO Follow up 2 years	Retrospective cohort study N = 32 COPD with *Pa* chronic bronchial infection	Reduction in exacerbations COPD by 38% *Pa* eradication of 28% at 2 years
Levofloxacin	Sethi et al 2012 [56]	240 mg BD via nebuliser for 5 out of every 28 days for 9–12 cycles	Double-blind randomised placebo-controlled trial N = 322 COPD with recurrent exacerbations randomised 2:1 ratio intervention:placebo	No difference in exacerbation rate or time to exacerbations

Studies on nebulised antibiotics have reported some controversial results and inconclusive data on their efficacy in other obstructive, suppurative lung diseases such as cystic fibrosis [49, 50] and non-cystic fibrosis bronchiectasis [51, 52]. Inhaled delivery of pharmacological therapy allows targeted drug delivery to the respiratory tract, thus reducing the systemic side effects of antimicrobial therapy and is potentially more suitable for prophylaxis. The most common side-effects are wheezing and local irritation, which can often be ameliorated with bronchodilator therapy prior to their use.

Despite these potential benefits, there are limited studies suggesting a benefit of inhaled antibiotics in the COPD population to date, which is summarised in Table 2 [53–56]. Most of these trials are small, single centre, uncontrolled studies and target those with *Pa* colonisation. *Pa* colonisation is an important prognostic factor in patients with COPD, as exacerbations in this setting are commonly challenging to treat, often requiring in-hospital management. *Pa* is notoriously difficult to eradicate and its presence can herald faster disease progression and higher mortality [57, 58]. Interestingly, there is huge variability in reported prevalence of *Pa* colonization rates in the above mentioned studies, 4.2% of over 22,000 outpatients and 40% of a much smaller cohort study of 18a outpatients, suggesting high variability within COPD populations. More severe exacerbator subgroups of patients will be associated with higher rates of *Pa* and other gram-negative organisms [59].

A randomised placebo controlled RCT by Sethi et al. evaluated the role of inhaled levofloxacin in COPD patients, irrespective of sputum pathogens [56]. Levofloxacin has a broad spectrum of activity against Gram-positive and Gram-negative aerobic bacteria and atypical bacteria, but limited activity against most anaerobic bacteria. This theoretically would be a reasonable choice for inhaled prophylaxis as the majority of pathogenic organisms in COPD would be covered. Compared with inhaled eradication strategies for resistant organism such as Pa that rotate four weeks of antibiotics, the authors feel that the 5 day regime each month, adopted in this study may have been inadequate to show a definitive benefit and trials of longer duration of therapy would be welcomed to clarify this limitation.

## Respiratory microbiome in patients with COPD

The role of the microbiome in the development and functional integrity of the immune system and colonisation resistance is increasingly recognised with key discoveries in the human microbiome project highlighting the importance and richness of bacterial microbiota in particular [60, 61]. The lung microbiota consists of the complete collection of microorganisms residing in the airways and parenchymal tissues of the lungs. Microbiome diversity is important for health including benefits in colonisation resistance, integrity of the epithelial cells and immunoregulation as supported by studies in gut microbiome.

An early observational cohort study by Gump et al. assessed the role of micro-organisms in 25 patients with chronic bronchitis over a four year period [62]. One third of the 116 exacerbations that occurred were possibly caused by viral infections. Viral infections were more commonly detected during exacerbations but also present to a lesser degree in stable states.

An observational cohort study by Erb-Downward et al. evaluated the bacterial microbiome in both current and non-smokers [63]. The authors used pyrosequencing of 16S amplicons to evaluate bronchoalveolar lavage to sample distal bronchi and air-spaces of 14 subjects as well as discrete tissue samples in six subjects. They found no significant difference in the overall heterogeneity of the bacterial community between nonsmokers, healthy smokers and COPD patients. Core respiratory bacteria microbiome included *Pa, Streptococcus, Prevotella, Fusobacterium, Haemophilus, Veillonella* and *Porphyromonas.* This study also found that lung microbiome diversity is lower in those with decreased lung function, and *Pa* more dominant in this group. Interesting, there were notable differences in the microbiome diversity of different anatomical regions of the lungs within patients with advanced COPD.

A further observational cohort study by Huang et al. used bacterial 16S to assess bacterial composition of endotracheal aspirates of intubated patients with severe COPD [64]. Two distinct bacterial populations were found; one group demonstrated a loss of diversity while a second group showed increased diversity, particularly the *Firmicutes* species. Previous studies by Hilty et al. and Sze et al. also correlated the presence of increased colonisation of *Firmicute*s with more advanced COPD [65, 66].

Pragman et al. performed a cross-sectional analysis of lung microbiota using BAL samples in patients with moderate to severe COPD and control subjects with respect to GOLD severity classifications [67]. The authors finding indicated those with COPD demonstrate early changes in lung microbiome even at an early stage and the use of inhaled medications such as corticosteroids and bronchodilators were associated with variance in bacterial colonisation.

In combination, these studies suggest that changes in the lung microbiome inn patients with COPD occur early in the disease process and although there is wide heterogeneity of organisms detected *Pa* and *Firmicutes* are shown to have increased representation in patients with COPD, particularly when at an advanced stage in their illness. However, it is important to note that these studies have not been consistently replicated, possibly due to differences in study design, methods of detection and analysis and anatomical differences between tissue samples and bronchoalveolar lavage.

The role of the respiratory microbiome in exacerbations of COPD is an important consideration given the morbidity and mortality associated with repeated exacerbations.

The majority of AECOPD are felt to be infectious in aetiology, triggered by acute bacterial and/or viral infections [3]. Sethi et al. reported that exacerbations are caused by new strains of bacteria being introduced to the host rather than new families or genera, in particular new strains of *Haemophillus influenza* were implicated [68]. The GOLD guidelines recommend the addition of the macrolide antibiotic azithromycin (provided no contraindications exist) as a thrice weekly or daily prophylactic treatment in addition to standard pharmacological management. RCT evidence from Albert et al.demonstrated a reduction in exacerbation frequency of 25% when administered for a 12 month period to patients with COPD [69]. How azithromycin works has not been fully elucidated but is more likely anti-inflammatory than directly antimicrobial. One theory is that the benefits of azithromycin are as a result of altered lung microbiome interaction with the host immune system. Multiple effects on the structure and composition of lower airway microbiota increase the levels of several microbial metabolites in the lung that have anti-inflammatory effects.

Observational cohort studies by Fodor et al. and Tunney et al. reported no significant change in lung microbiota during exacerbations of other respiratory conditions such as cystic fibrosis and bronchiectasis [70, 71]. However, when bacterial sequencing analysis, small changes in bacteria were noted following antibiotic usage in some patients compared to their baseline sequencing.

Wang et al. assessed dynamic changes of lung microbiome during COPD exacerbations by evaluating 16S ribosomal RNA on the lung microbiome of 87 patients with COPD during stable state, time of exacerbation, two weeks post-treatment and at six weeks recovery [72]. The authors reported dynamic changes within the lung microbiota associated with exacerbation events. They found that antibiotic and steroids treatment were associated with changes in lung microbiome and certain biomarkers including interleukin-8 are highly correlated with microbiome diversity. Additionally, disturbance of bacterial units such as *Haemophilus* species could have a significant impact on the microbial community.

A subsequent observational study by Sun et al. compared 16 s ribosomal DNA sequencing obtained from AECOPD patients during exacerbation at day 1, 7 and 14 [73]. At all three time-points, the abundance and diversity of the flora in the acute phase changed significantly compared with stable states (p = 0.001). Individual changes in specific bacterial species were non-significant apart from Acinetobacter which increased during exacerbations compared to stable states (p = 0.022).

## Gut-lung microbiome

There is increasing evidence evolving to support a close relationship between the gastrointestinal and respiratory microbial ecosystem and the association between changes in the gut-lung microbiome and exacerbations of COPD. The gut-lung axis appears to have an important role in shaping local cell function, directing host immune responses within and outside the lung, and chronic disease development [74, 75] .

Chronic lung conditions, such as COPD, often occur in tandem with chronic disorders of the gastrointestinal tract such as inflammatory bowel diseases and functional bowel disorders [76]. Patients with COPD are two or three times more likely to develop IBD compared with the general population. Observational studies have demonstrated increased intestinal permeability in patients with COPD compared with matched healthy controls [77]. Furthermore, many patients with COPD, particularly those with frequent exacerbations, are prescribed multiple courses of antibiotics and corticosteroids as part of their acute AECOPD treatment. This can result in dysregulation of the gut microbiome and promote antibiotic resistance and proliferation of pathogenic organisms.

Sun et al. also compared the microbiome of gut profiles with lung samples in patients with AECOPD during the acute, treatment and stable states [73]. Firmicutes and Bacteroidetes were more frequent in gut samples, while Acidobacteria and Cyanobacteria were much lower. While the abundance of bacteria differed over time with decreasing presence from acute states to stable states, the diversity did not significantly change between disease states.

Gut bacteria can travel to the respiratory tract through a number of mechanisms, aspiration of gastric contents or gastro-oesophageal reflux disease. GORD is prevalent in COPD populations with 26% of patients with COPD reporting symptoms of GORD in the ECLIPSE longitudinal observational study [78]. A positive association with GORD and frequency of exacerbations has also been reported [79, 80]. Furthermore, any disturbance in the integrity of the respiratory epithelium can allow translocation of bacteria to enter the systemic circulation. The presence of chronic periodontitis is associated with increase in exacerbations of COPD, likely due to a combination of factors including micro-aspiration, translocation of pathogenic oral bacteria to the respiratory tract [81–83]. This is potentially an important under-recognised causal or at least an associated factor in recurrent AECOPD.

There is recent interest in the benefits of dietary fibre in providing local and systemic anti-inflammatory effects and repair the gut dysbiosis. Research focusing on the relationship between dietary patterns and lung function in patients with COPD suggests that a prudent diet high in dietary fibre may be protective in COPD [84].

The use of live microorganisms in the form of probiotics to promote restoration of the intestinal microbiome has shown inconsistent results in studies in asthma [85–87]. There are no human studies on the utility of probiotic usage in patients with COPD although a study in the effect if prebiotics and prebiotics were found to reduce alveolar damage in mice models [88].

Recent evidence has highlighted important associations between OSA and the gut microbiome a bidirectional relationship between OSA and microbiota composition has been suggested. Sleep fragmentation, intermittent hypoxia, and intermittent hypercapnia all play significant roles in altering the microbiome, and initial evidence has shown that alterations of the microbiota affect sleep patterns.

Dysbiosis in the gut microbiome in the setting of OSA has been demonstrated in animal studies [89]. A further cohort study in paediatric populations with OSA using16S rRNA gene sequencing indicated that the oral microbiome composition was significantly disturbed in paediatric OSA compared with normal controls, especially with regard to *Firmicutes*, *Proteobacteria*, *Bacteroidetes*, *Fusobacteria*, and *Actinobacteria* [90].

Pre- and probiotics, short chain fatty acids and faecal matter transplantation may offer potential as novel therapeutic strategies that target this gut dysbiosis, however studies evaluating these novel therapies are in early stages of preclinical and clinical development [91].

The current literature provides evidence of associations of antibiotics and other treatments such as corticosteroids and proton pump inhibitors with dysregulation of the gut-lung microbiome. Antimicrobial stewardship plays an important role in determining the necessity for antibiotic prescribing and also to guide the ideal duration depending on individual host factors.

## Areas identified for further research

Taking into account the available literature to date and the authors’ clinical experience, there are a number of areas where focused research is desirable to best inform clinical recommendations for patients who continue to exacerbate despite adherence to best practice guidelines:

1. Identification of clinical phenotypes and co-morbid conditions: Large prospective studies are needed to identify clinical phenotypes of high frequent exacerbators, their functional status and to identify and treat comorbidities which can predispose to AECOPD such as GORD, aspiration events, rhinosinusitis, OSA and periodontitis
2. Identification of patients with risk factors for colonisation due to impaired airway clearance: Given the prevalence of co-existent bronchiectasis with COPD, large multicentre studies that evaluate the use of HRCT to assess for evidence of bronchiectasis and mucus plugging in addition to interventional arms to assess the effect of airway clearance techniques, PEEP-based therapies and extended courses of antimicrobial therapy are advisable [92]
3. Interventional studies of alternative antimicrobial therapies: Convincing evidence for alternative antimicrobial therapies, including anti-viral therapies, is lacking in well-conducted, RCTs of adequate sample size. Further studies exploring the role of regular sputum analysis during stable states and exacerbations to assess for evidence of colonisation (e.g *Mycobacterium* species or *Pa*) and guide antimicrobial sensitivities are indicated.
4. Role of anti-viral measures: The role of strict infection control measures such as frequent handwashing, avoiding crowded areas and excessive social contacts, along with the wearing of face coverings particularly during winter months has promise in reducing AECOPD secondary to viruses and merits further dedicated study.

## Conclusion

COPD exacerbations are major global health concern as patients who frequently exacerbate have more symptoms, have more frequent and longer in-hospital stays, experience a decline in lung function and an abbreviated lifespan compared to non-exacerbators.

Current guidelines lack a comprehensive approach to manage patients who are frequently exacerbating despite adherence to best practice treatment. Our literature enquiry has demonstrated a paucity of well-conducted studies on alternative therapies in this setting. However, there are a number of approaches that may herald positive benefit for this patient population; clinical phenotyping which encompasses multimorbidity, identification and optimal management of co-existing conditions such as BE, GORD, OSA, rhinosinusitis and aspiration events, evaluation of lung and gut microbiome during exacerbations and stable state to guide antimicrobial therapies and appropriate stewardship and future development of anti-viral treatments particularly against rhinoviruses groups.

### Take home message

The majority of AECOPD are triggered by infections however specific long term antimicrobial therapies are limited. We review the literature regarding ‘off guideline’ antimicrobial trials in exacerbation-refractory COPD patients.

## Data Availability

All data described in the manuscript is freely available. This manuscript does not describe any new software, databases and all relevant raw data that is not already available from the appended references.

## Abbreviations

AECOPD: Acute exacerbation of chronic obstructive pulmonary disease
BAL: Bronchoalveolar lavage
BD: Twice daily
BMI: Body mass index
CD: Cluster of differentiation
COPD: Chronic obstructive pulmonary disease
CPAP: Continuous positive airway pressure
FEV1: Forced expiratory volume in 1 second
GOLD: Global initiative for chronic lung disease
GORD: Gastro-oesophageal reflux disease
HRCT: High resolution computed tomography thorax
ICS: Inhaled corticosteroid
IL: Interleukin
IU: International units
LABA: Long acting beta 2 agonist
LAMA: Long acting muscarinic agonist
Mg: Milligrams
Ml: Millilitre
N: Number
NNT: Number needed to treat
OSA: Obstructive sleep apnoea
PA: *Pseudomonas aeruginosa*
PCR: Polymerase chain reaction
PEEP: Positive end expiratory pressure
RCT: Randomised control trial
RNA: Ribonucleic acid
RSV: Respiratory syncytial virus
Vacc: Vaccine
